# Presence of Two Separate Hairy Polyps with Meningothelial Elements in a 15-Month-Old Child

**DOI:** 10.1155/2021/1844244

**Published:** 2021-09-21

**Authors:** Ahmad Saeed A. Alghamdi, Nasser Almutairi, Ghassan Alokby

**Affiliations:** ^1^College of Medicine, Taif University, Taif, Saudi Arabia; ^2^Department of Otolaryngology—Head and Neck Surgery, King Faisal Specialist Hospital and Research Centre, Riyadh, Saudi Arabia; ^3^Department of Otolaryngology—Head and Neck Surgery at College of Medicine, Alfaisal University, Riyadh, Saudi Arabia; ^4^Clinical fellowship in Rhinology and Paranasal Sinus Surgery at the University of Iowa Hospital and Clinics, Iowa City, IA, USA; ^5^Clinical fellowship in Rhinology and Endoscopic Skull Base Surgery at the University of Miami Miller School of Medicine, Miami, FL, USA

## Abstract

Hairy polyps are benign embryological tumors of the head and neck region that are derived from two germinal layers, the ectoderm and mesoderm. At an incidence of 1 : 40000, hairy polyps are considered rare growths. Presenting symptoms of hairy polyps can vary greatly depending on the size and location of the tumor. To the best of our knowledge, our reported case is the first in the literature to highlight an extremely rare occurrence of two separate hairy polyps being simultaneously present in one patient, in the clivus and the nasion, with the presence of meningothelial cells within both tumors, histopathologically. With surgical resection as the management of choice, the approach of surgery differs greatly depending on many factors. Since the patient in our reported case had a cleft palate, we used a combined transnasal and transoral approach to fully release the clival mass and a direct skin incision for the nasion mass. Recurrence after complete surgical resection is rare, and if happens, it should rise suspicion of histopathologically misdiagnosed tumor. We amplified the importance of advanced radiological investigations along with proper multidisciplinary teamwork to exclude CNS connections and other histologically malignant tumors and to early pick up a possible simultaneous lesion.

## 1. Introduction

Hairy polyps are benign growths that originate from two germinal layers, the ectoderm and mesoderm. Being first described by Brown-Kelly in 1918, hairy polyps are embryological malformations that usually present in the early childhood period. Hairy polyp is a rare tumor in the head and neck, with an incidence of 1 case in every 40,000 individuals. We reported a unique case of two separate hairy polyps with meningothelial cells occurring simultaneously in one patient.

## 2. Case Presentation

15-month-old male baby, a product of an uneventful dizygotic pregnancy with his twin brother being totally normal, was delivered by cesarean section due to obstetric reasons. At the age of 7 days, he was admitted in another hospital for the excision of a tongue dermoid cyst prior to referral to our center. The patient was referred to KFSHRC primarily for the evaluation of an incomplete cleft palate with a tongue mass. On examining the patient, a cleft palate was noted with a midline hard tumor at the posterior edge of a bifid palate at a size of around 2.5 × 1.5 cm, along with a residual tongue lesion measuring 2 × 1.5 cm at the anterior dorsal surface of the tongue.

**Figure 1:**
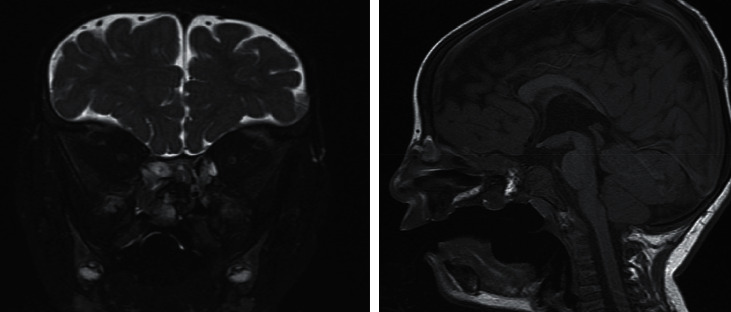
MRI of the head and neck showing right nasal mass and nasion mass which is hypointense in T1 and isointense in T2.

The patient was planned to have MRI (Figure 1) that showed a midline palatal defect involving both hard and soft palates, along with what appears to be a polypoidal mucosal mass with retention cysts protruding through defect from the right nasal cavity to the palate posteriorly. The radiologist then suggested CT facial bones to be done.

**Figure 2:**
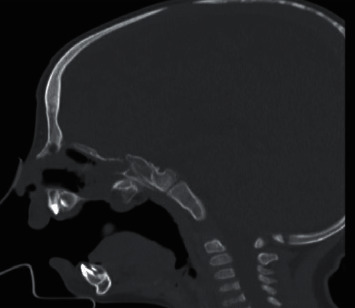
CT facial bones: cleft lip and palate with approximately 10 mm gap within the hard palate and prominent posterior bone vomer with no skull base defect.

On the next visit, CT facial bones were done and showed cleft lip and palate with other findings, as shown in Figure 2.

The impression, based on radiological findings and clinical examination, was a skull base mass at the clivus, extending to the sphenoid sinus. In addition to that, the patient had a midline nasal mass in the area of the nasion with extension to the skin but without any sinus.

Given that the patient had a cleft palate, the decision was made to perform combined endoscopic transnasal and transoral resection of the clival lesion with resection of the midline nasal structure. Informed consent for the surgery was then taken from the family.

The patient was taken to the operation room. Nasal examination under general anesthesia using a 30-degree scope was done for the first time, and we were able to identify a clival hard lesion that was extending through the cleft into the oral cavity. Access to both sides of the lesion was achieved by a small posterior septectomy, lateralizing the inferior turbinate and incising the posterior septum. Using a diamond drill, the lesion was drilled off the clivus. And by using a true cutting instrument, all soft tissue attachments were released intranasally, superiorly, medially, and laterally. The remaining attachment was an anterior attachment to the floor of the nose, which was released by a transoral approach. At this stage, en block resection of the mass was achieved.

For the other mass over the nasion, a midline incision was done over the nasion. After elevating the periosteum, a cyst was identified with resultant cavitation in the bone. The cyst was dissected completely, and the cavity was drilled using a diamond drill to remove any remaining soft tissue. The incision was then closed in two layers.

Postoperatively, the patient was doing fine and tolerating oral feeding. After 24 hours, he was discharged with a clinic appointment.

**Figure 3:**
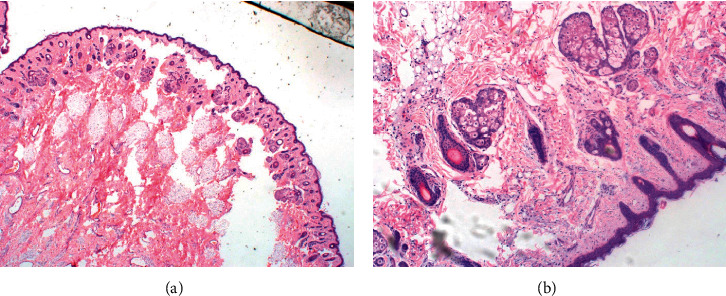
H&E-stained section at 2x (a) and 10x (b) showing polypoid lesion with keratinizing skin surface epithelium. Meningothelial cells are noted along with dermal adnexal structures with a core of fibroadipose tissue.

On histopathological examination, grossly, the clival mass was white-tan irregular firm tissue measuring 2 × 2 × 1 cm, with homogeneous white nodular cut surface. The midline nasal mass was white-tan hemorrhagic fibrous tissue, measuring 2 × 2 × 0.3 cm. So, both masses showed the presence of teratoid hairy polyps with meningothelial cells and tooth tissue noted. No endodermal elements were seen on the submitted specimen, as shown in Figure 3.

Regular follow-ups with CT scans showed no progression in the teratoid over a period of 18 months postoperatively. Given that he is asymptomatic with no clinical signs of recurrence, the decision was elected to observe the patient on serial imaging keeping in mind that the patient may need surgical intervention through an endoscopic or craniotomy approach in the future depending on the symptoms and any progression. The parents were counselled in detail regarding their child's condition.

## 3. Discussion

Oropharyngeal and nasopharyngeal hairy polyps, although not common, but are well described embryological malformations in the literature. The first case of hairy polyp was reported by Brown-Kelly in the early twentieth century, 1918 [1]. These malformations are the result of totipotential cells misplacement that give rise to such outgrowths [2]. Hairy polyps are another name of dermoids, which are one of four categories Arnold et al. used to classify oropharyngeal and nasopharyngeal malformations back in 1888. The four classes are dermoids arising from the epidermal and mesodermal germinal layers, teratomas originating from all three germ layers, teratoids, a poorly differentiated form of teratomas, and epignathi, the most differentiated class, represents a parasitic fetus, with development of limbs and other organs inside the tumor [3]. Schwalbe, in 1907, came up with another list of four categories: extremely rare pharyngeal masses that are composed of well-differentiated elements, masses that have well-differentiated organ-like structures on gross examination, teratomas, and dermoids. Hairy polyps are commonly to be mistakenly called teratomas, which arise from all three germinal layers, while hairy polyps originate from ectoderm and mesoderm only [4]. Hairy polyps are still a rare tumor with a female predominance of 6 : 1 male, and only around 180 cases are reported in the literature with an incidence of 1 in 40,000 [5, 6]. Hairy polyps can be located anywhere, but 60% of these lesions originate from the nasopharynx or the superior aspect of the soft palate [2, 7]. Other locations include both soft and hard palates, tonsillar areas, middle ear cavity, eustachian tube, and even the tongue [5]. Focusing on the laterality of hairy polyps, most of the lesions reported were located in the left side [8]. However, Yilmazer et al. reported the first case of simultaneous bilateral hairy polyps in a female newborn in 2015 [9]. Symptoms caused by hairy polyps vary greatly depending on the size and location of the lesion, from being relatively asymptomatic, like our reported case, to those causing a wide range of symptoms ranging from suffocation and neonatal stridor to feeding difficulties, vomiting, hemoptysis, and recurrent middle ear infections [10, 11]. Hairy polyps are not considered as a representation of any syndrome but are occasionally associated with other malformations such as cleft palate like our case, ankyloglossia, hemihypertrophy of the face, uvular agenesis, and different other malformations in areas other than the head and neck [12]. The differential diagnoses of hairy polyps include hamartoma, neuroblastoma, glioma, meningoencephalocele, and other masses found in early years of life [13]. These many possible diagnoses cannot be differentiated on regular radiological films. In order to be confident having an exact preoperative idea about the lesion, CT and MRI are usually used to narrow down the possibilities. For instance, the presence of fat tissue characteristics on these imaging modalities suggests the diagnosis of either hairy polyp, teratoma, or hamartoma [13]. When cystic areas and calcifications are present, hairy polyp is less likely compared to teratoma [14]. Out of these three fat-containing masses, hairy polyps are the most common [2]. In addition to that, CT and MRI were utilized in our reported case to exclude any connection with the central nervus system, as a defect was present at the clivus. The management of choice in hairy polyps is surgical excision [15]. However, the approach of surgery differs greatly depending on the location of the lesion. The use of endoscopy to excise nasopharyngeal hairy polyps is a safe choice that provides good view and control in the surgical field [16]. In our case, we used a combined transnasal and transoral approach to fully release the clival mass. For the nasion lesion, a direct incision over the area was the best to excise it. The histopathological features of hairy polyps are mesodermal structures such as adipose, fibroconnective, and lymphoid tissues with minor salivary glands, cartilaginous, bony, and muscular cells. The ectodermal component of hairy polyps is composed of keratinized squamous epithelium with other skin appendages such as hair, sweat, and sebaceous glands [17]. Meningothelial cells are the building blocks of the meninges, the protective layers covering the central nervous system [18]. It is rare for meningothelial cells to be found within another benign tumor in the head and neck [19]. When found extracranially, meningothelial proliferations can be classified into four categories: extracranial extensions of an intracranial meningioma, extracranial meningiomas, metastatic meningiomas, and primary cutaneous meningiomas [7]. In the literature, Olivares-Pakzad et al. reported the only case of hairy polyp with meningothelial elements. On the other hand, the simultaneous presence of two hairy polyps in the same patient is extremely rare, with only one reported case by Simoni et al. in 2003 where he presented a case of a one-day-old female who underwent resection of a posterior pharyngeal mass that was diagnosed as a hairy polyp pathologically. When 10-month-old, the same patient was discovered to have a salivary gland choristoma in the middle ear [20]. To the best of our knowledge, our case is the first reported in literature to have two simultaneous hairy polyps in two different locations, with each of them having meningothelial elements. There are many theories to explain the presence of these cells within hairy polyps. The inclusion theory suggests that arachnoid cells from cranial and peripheral nerves became imbedded in deeper layers of the tissue, thus becoming an obstacle for normal fusion to occur and resulting in, for example, cleft palate [7]. Given the benign characteristics of hairy polyps, recurrence is rare after complete surgical excision, and if happens, it should rise suspicion that the tumor was initially misdiagnosed histopathologically [7]. However, a recurrence is possible as reported by Chang et al. and Haddad et al. [15, 21].

## 4. Conclusion

Hairy polyps of the oronasopharynx are benign growths that may present with a wide range of symptoms, depending on size and location. In our case report, we amplify the importance of advanced radiological investigations along with proper multidisciplinary teamwork to exclude CNS connections and to early pick up a possible simultaneous lesion.

## Data Availability

The data used to support the findings of this study are available from the corresponding author upon request. For privacy of the patient, the data are not publicly available.
